# The Urine Metabolome of Young Autistic Children Correlates with Their Clinical Profile Severity

**DOI:** 10.3390/metabo10110476

**Published:** 2020-11-23

**Authors:** Michele Mussap, Martina Siracusano, Antonio Noto, Claudia Fattuoni, Assia Riccioni, Hema Sekhar Reddy Rajula, Vassilios Fanos, Paolo Curatolo, Luigi Barberini, Luigi Mazzone

**Affiliations:** 1 Department of Surgical Sciences, School of Medicine, University of Cagliari, 09042 Monserrato, Italy; hemasekhar09@gmail.com (H.S.R.R.); vafanos@tiscali.it (V.F.); 2 Department of Biomedicine and Prevention, Tor Vergata University of Rome, 00133 Rome, Italy; siracusanomartina@hotmail.it; 3 Department of Biotechnological and Applied Clinical Sciences, University of L’Aquila, 67100 L’Aquila, Italy; 4 Department of Medical Sciences and Public Health, University of Cagliari, 09042 Monserrato, Italy; antonionoto@unica.it (A.N.); barberini@unica.it (L.B.); 5 Department of Chemical and Geological Sciences, University of Cagliari, 09042 Monserrato, Italy; cfattuon@unica.it; 6 Child Neurology and Psychiatry Unit, System Medicine Department, Tor Vergata University Hospital of Rome, 00133 Rome, Italy; assiariccioni@gmail.com (A.R.); curatolo@uniroma2.it (P.C.); luigi.mazzone@uniroma2.it (L.M.)

**Keywords:** autism spectrum disorder, metabolomics, hierarchical clustering analysis, gut dysbiosis, food selectivity, autism core deficits

## Abstract

Autism diagnosis is moving from the identification of common inherited genetic variants to a systems biology approach. The aims of the study were to explore metabolic perturbations in autism, to investigate whether the severity of autism core symptoms may be associated with specific metabolic signatures; and to examine whether the urine metabolome discriminates severe from mild-to-moderate restricted, repetitive, and stereotyped behaviors. We enrolled 57 children aged 2–11 years; thirty-one with idiopathic autism and twenty-six neurotypical (NT), matched for age and ethnicity. The urine metabolome was investigated by gas chromatography-mass spectrometry (GC-MS). The urinary metabolome of autistic children was largely distinguishable from that of NT children; food selectivity induced further significant metabolic differences. Severe autism spectrum disorder core deficits were marked by high levels of metabolites resulting from diet, gut dysbiosis, oxidative stress, tryptophan metabolism, mitochondrial dysfunction. The hierarchical clustering algorithm generated two metabolic clusters in autistic children: 85–90% of children with mild-to-moderate abnormal behaviors fell in cluster II. Our results open up new perspectives for the more general understanding of the correlation between the clinical phenotype of autistic children and their urine metabolome. Adipic acid, palmitic acid, and 3-(3-hydroxyphenyl)-3-hydroxypropanoic acid can be proposed as candidate biomarkers of autism severity.

## 1. Introduction

The prevalence of autism spectrum disorder (ASD), a pervasive brain-based developmental disorder, is abruptly increasing worldwide [1,2]; in 2020, the Autism Developmental Disabilities Monitoring (ADDM) Network estimated that 1 in 54 children are affected by ASD in the United States [3]. The associated public health impact implies not only dramatic consequences on affected children and their parents/families, but also growing financial costs due to expenditures for medical care, intensive behavioral intervention, and loss of productivity for parents and for the affected people [4]. Although ASD is highly heritable, robust evidence has revealed a powerful genome-environment interplay [5]; additionally, specific changes in the gut microbiota composition are emerging as key factors in ASD pathogenesis [6]. Thus, ASD diagnosis is expected to move from the identification of common inherited genetic variants to a system biology approach, based on the detection of the individual molecular phenotype. As metabolites are the building blocks of the molecular phenotype, metabolomics has emerged as a multidisciplinary science assessing the whole set of low molecular mass molecules within a biological matrix, namely the metabolome [7]. Since 2010, several studies have shown that autistic subjects might share metabolic abnormalities linked with amino acid and purine metabolisms, energy production, oxidative stress, and gut microbiota fermentation of nutrients and toxicants [8]. Our aims were: to explore the most relevant metabolic perturbations in ASD; to investigate whether the severity of ASD core symptoms, evaluated by gold standard diagnostic instruments, may be associated with urine metabolic alterations; and to examine whether the urine metabolome discriminates severe from mild-to-moderate restricted, repetitive, and stereotyped patterns of behavior, quantified by standardized behavior rating scales filled out by parents.

## 2. Results

The clinical and behavioral examinations of neurotypical (NT) children (median age: 4.46 years; interquartile range: 3.25–5.0 years) confirmed this group as controls. In ASD children (Table 1), 19.4% exhibited gastrointestinal disease; 55% had food selectivity.

Overall, 154 metabolites were recognized in the urine of ASD children and controls by comparing retention times, and mass spectra with those stored in an in-house made library including more than 255 metabolites obtained injecting pure standard compounds. Eleven metabolites (7.1%) were excluded because of: missing data during analysis or unreliable data or due to interfering factors partially or wholly altering the final result (Table S1); 25 metabolites (16.2%) were identified by their mass and chromatography retention time; however, their biochemical identity was unknown, and thus they were ruled out as well (Table S2).

### 2.1. Urine Metabolic Profile in ASD Children and NT Children

**Figure 1:**
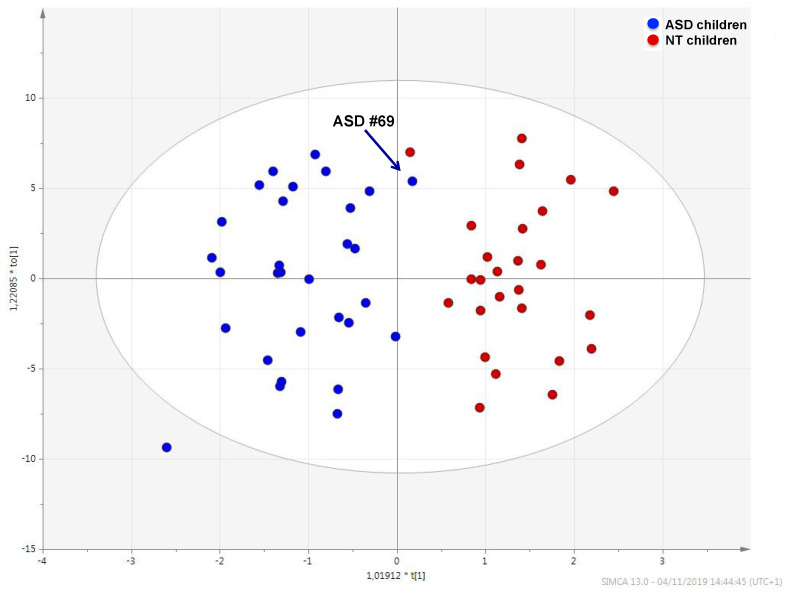
OPLS-DA score plot showing the metabolic profile of ASD children (blue dots) and that of typically developing children (red dots). Black arrow indicates the metabolome of ASD child #69.

The urinary metabolic profile of children with ASD was largely distinguishable from that of NT children (Figure 1); the quality of this model was represented by R^2^ X = 0.525, R^2^ Y = 0.808, Q^2^ = 0.609 and confirmed by analysis of variance CV-ANOVA (*p* < 0.0001).

Sample #69, marked in the plot by an arrow, fell within the metabolic cluster of NT children. This sample belongs to of a six-years-old autistic child with neither food selectivity nor gastrointestinal disease; calibrated scores obtained by observational assessments and questionnaires were below cut-off limits and median values (Table 2). The receiver operating characteristic (ROC) analysis yielded an AUC = 0.892 (95% confidence interval—C.I.: 0.724–0.996) (Figure S1).

Univariate and multivariate statistical analysis identified 13 metabolites as those best discriminating ASD from NT children (Table 3). Differences were found between a subgroup of 17 ASD children (55%) with food selectivity (or picky eating) and the remaining ASD children eating a balanced variety of food.

**Figure 2:**
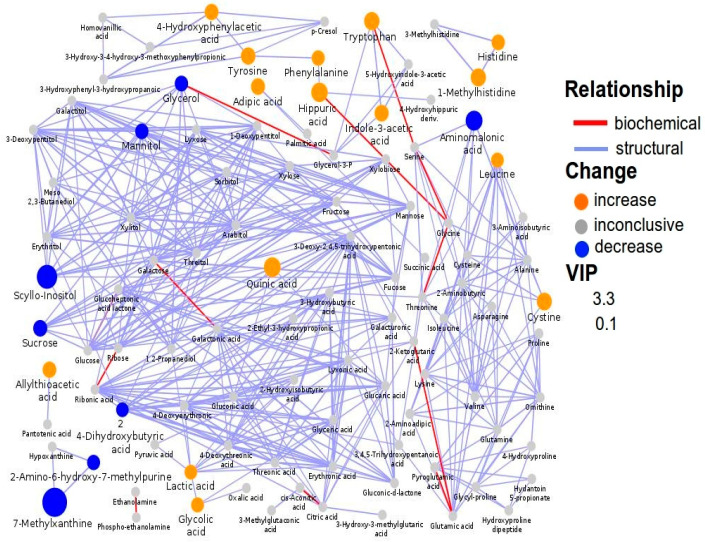
Biochemical network mapping resulting from the comparison between the urine metabolome of ASD children and that of NT children. Metabolites connections are based on biochemical (red edges) and structural (violet edges) similarities. Structural similarity is shown for Tanimoto coefficients (≥0.7, solid edges) and relaxed scores (dashed edges). Node size displays model VIP and color the direction of change in ASD relative to control samples.

Although 7-methylxanthine was significantly decreased in ASD children compared to NT children, the decrease was less pronounced in the subgroup of ASD children with food selectivity (*p* = 0.043). Network mapping analysis showed a significant metabolic shift (Figure 2); the resulting relationships, based on the number of interconnected metabolites, indicated several metabolites strongly associated each other, such as 1-methylhistidine with histidine; scyllo-inositol–mannitol–glycerol (osmoregulation); 4-hydroxyphenyl acetic acid–tyrosine–phenylalanine–hippuric acid (gut metabolism of tyrosine); tryptophan with indole-3-acetic acid (gut metabolism of tryptophan); and 7-methylxanthine with 2-amino-6-hydroxy-7-methylpurine (purine metabolism).

### 2.2. Association between ASD Core Symptoms and the Urine Metabolome

The urine metabolic profile of children having an Autism Diagnostic Observation Schedule–Second Edition (ADOS-2) with calibrated severity score (CSS) ≥ 8 (56%, corresponding to severe deficits in social affect and ritualistic repetitive behavior) was marked by the significant increase of metabolites derived from diet (e.g., sucrose, xylose), gut dysbiosis (e.g., *p*-Cresol, hippuric acid, allantoin), perturbations of the tryptophan metabolism (e.g., quinolinic acid, 5-hydroxyindoleacetic acid), and mitochondrial dysfunction (adipic acid). The urine metabolic profile of this subgroup was distinguishable from that of ASD children with an ADOS-2 CSS < 8 (Figure S2); 22 metabolites were those better discriminating the two subgroups of autistic children (Table 4). Ten metabolites significantly correlated with the ADOS-2 CSS score (Table S3).

**Figure 3:**
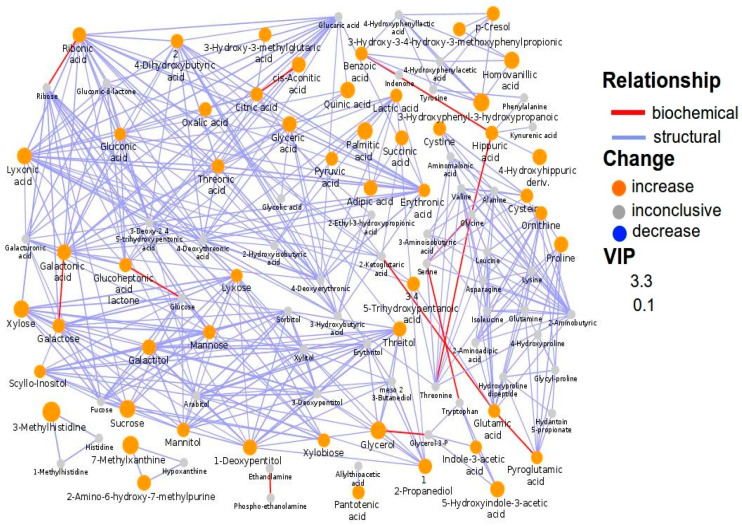
Biochemical network mapping resulting from the comparison between the urine metabolome of ASD children with ADOS-2 CSS ≥ 8 and that of ASD children with ADOS-2 CSS < 8. Metabolites connections are based on biochemical (red edges) and structural (violet edges) similarities. Structural similarity is shown for Tanimoto coefficients (≥0.7, solid edges) and relaxed scores (dashed edges). Node size displays model VIP and color the direction of change in ASD relative to control samples.

Quinic acid and hippuric acid were significantly increased in ASD children; however, in the subgroup with food selectivity, the increase of quinic acid (+263%) was much more pronounced than that of hippuric acid (+164%). Indeed, quinic acid and hippuric acid were significantly different between the two subgroups (*p* = 0.027 and *p* = 0.017, respectively). Network mapping highlighted various relevant interconnections: adipic acid with succinic acid, reflecting mitochondrial dysfunction; 3-(3-hydroxyphenyl)-3-hydroxypropanoic acid (HPHPA) with *p*-cresol and benzoic acid with hippuric acid, both reflecting *Clostridia spp.* overgrowth; adipic acid with palmitic acid and sugars as well as lyxonic acid with 2α-ketoglutaric acid, reflecting the dietary style (Figure 3).

### 2.3. Association between Repetitive, Problematic Abnormal Behaviors and the Urine Metabolome

**Figure 4:**
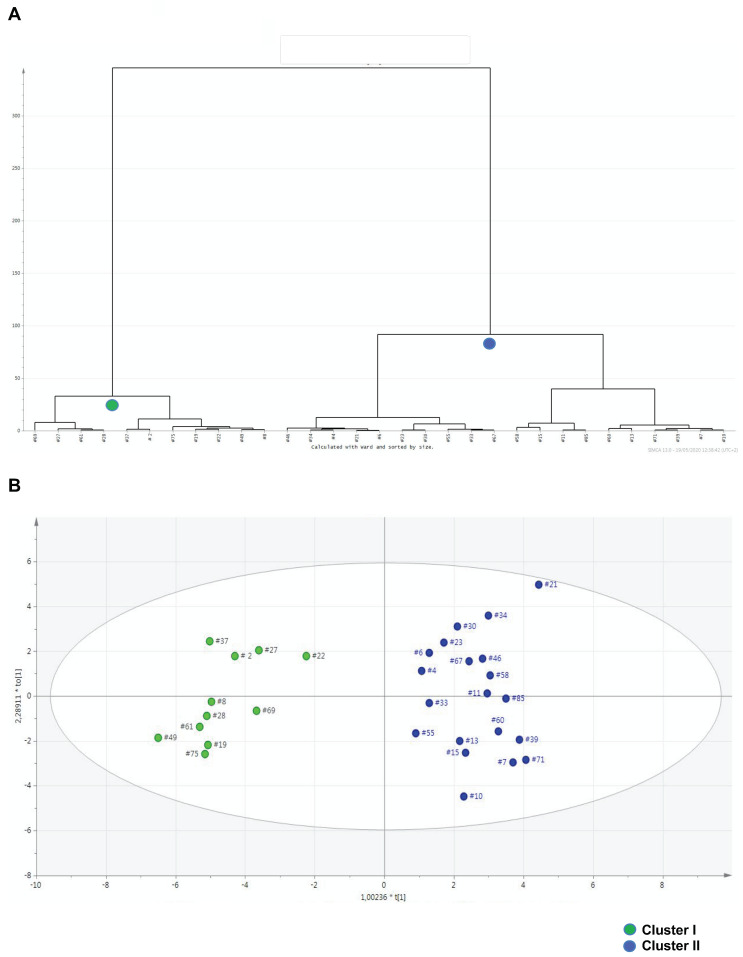
Hierarchical cluster analysis (HCA) represented by the dendrogram plot (**A**); the horizontal lines depict the grouping of clusters and the distance between two joining clusters, the vertical lines represent the differences of these distances. OPLS-DA scatter plot (**B**) of the first principal component obtained from GC-MS spectra of urine samples from cluster I (green dots, n = 11) and cluster II (blue dots, n = 20). Both dendrogram and OPLS-DA scatter plot report the identification number for each urine metabolome. Clusters I and II were obtained by HCA, as depicted by the dendrogram.

**Figure 5:**
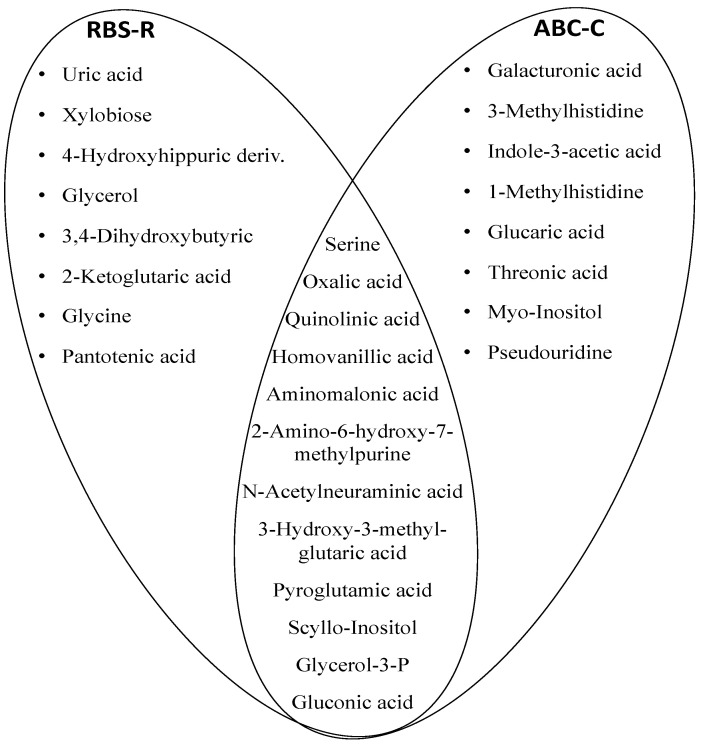
Venn diagram illustrating the top 25 metabolites shared between groups and discriminant ASD children with severe impaired behaviors from those with mild-to-moderate impaired behaviors. The visualization of the common metabolic content is suggestive to the presence of the perturbed pathways such as oxidative stress, gut dysbiosis and purine metabolism. The most perturbed pathways shared by the two scales (RBS-R and ABC-C) were glycine, serine, and threonine metabolism, tryptophan metabolism, oxidative stress, mitochondrial energy supply, and purine metabolism.

By HCA, the urine metabolic profiles of 31 ASD children were grouped within homogeneous clusters by merging them one at a time in a series of sequential steps. Two significantly different clusters were finally obtained, i.e., the cluster I (11 urine metabolomes), and the cluster II (20 urine metabolomes) (Figure 4A). The corresponding OPLS-DA model was highly significant: R^2^ X = 0.464, R^2^ Y = 0.918, Q^2^ = 0.567 and *p* = 0.00024 (Figure 4B). We assumed this model as the reference model against which to compare differences in abnormal behaviors. Then, we built a data matrix (Table S4). In cluster II, most children exhibited a Repetitive Behavior Scale-Revised (RBS-R) score ≤ 35 and an Aberrant Behavior Checklist-Community (ABC-C) score ≤ 50; only three children (15%) had an RBS-R score above 35, and only two (10%) an ABC-C score above 50. Within cluster I, six children (54.5%) had an RBS-R score above 35, and seven (63.6%) had an ABC-C score above 50. Based on these findings, we preliminarily assumed RBS-R and ABC-C score thresholds 35 and 50, respectively. Finally, urine metabolome of children with RBS-R ≤ 35 was compared with that of children with RBS-R > 35; similarly, we compared ABC-C ≤ 50 versus ABC-C > 50 (Figure 5, Table 5). Even though the sizes of the RBS-R and ABC-C subgroups above and below the thresholds were equal (22 and 9), children were not the same.

## 3. Discussion

### 3.1. The Metabolic Profile of Autistic Children

Perturbations in several metabolic pathways shape the urine metabolome of ASD children; even though each ASD child exhibits an individualized urine metabolome, the group forms a homogeneous set, far different from that of NT children. The urine metabolome of sample #69 is placed very close to the set of NT children. Interestingly, sample #69 belongs to an autistic child with very mild impairment in reciprocal social interaction and restricted, repetitive or sensory behaviors (Table 2). Despite this, we refused to rule out this child from the group of ASD children, as the child was not eligible for the inclusion within the NT group.

In ASD children, 7-methylxanthine and uric acid were significantly decreased (Table 3), reflecting oxidative stress and specific dietary styles. Methylxanthines are largely synthesized in a restricted number of botanical species; conversely, their synthesis in humans is negligible [9]. The limited intake of 7-methylxanthine reduces a number of beneficial effects [10]. The diet is the main factor influencing the urinary excretion of 7-methylxanthine: high intakes of chocolate, cocoa, tea, and cola-based beverages lead to high urine excretion of 7-methylxanthine and vice versa [11]. Despite 7-methylxanthine was significantly reduced in ASD children compared with NT children, in the subgroup of ASD children with food selectivity the reduction was less pronounced. As a matter of fact, 7-methylxanthine was higher in the subgroup of ASD with food selectivity compared with that with balanced diet (+44%, *p* = 0.043). On the one hand, rigid and repetitive dietary patterns induce inadequate nutrient intake; on the other hand, food selectivity could imply an excess of cola beverages, non-cola soft drinks, chocolate, and ice cream [12,13].

In the group of ASD children, oxidative stress and gut dysbiosis induced several metabolic perturbations. First, uric acid was decreased and associated with an increase in hypoxanthine (+28%, *p* = n.s.) and allantoin (+43%, *p* = 0.03). This association may well be consistent with bacterial uric acid degradation by uricase, an enzyme not expressed in humans because of the presence of so-called “nonsense” mutations in the encoding gene [14,15]. Controversial results on uric acid in ASD have been previously published; in a very small subgroup of autistic children (nine), urinary uric acid was found significantly increased due to the considerable increase of de novo purine synthesis, as demonstrated by the in vitro culture of their skin fibroblasts [16]. More recently, two research groups found that autistic subjects compared with NT subjects exhibit reduced levels of urinary uric acid: the first study enrolled 48 autistic subjects with a mean age of 10.7 years (standard deviation 4.0 years) and the second one 90 autistic children, aged 1.5–8.0 years [17,18]. Further studies found significant increases of uric acid in the plasma of autistic children, as summarized in two recent reviews [8,19]. Beyond variability between studies, the biological role of uric acid within the CNS remains still questionable: from one hand, uric acid is neuroprotective, being an anti-oxidant factor [20]; on the other hand, excess of uric acid may be neurotoxic [21,22].

Oxidative stress seems to be largely involved in determining the significant increase in cystine in ASD children. Several factors contribute to increasing the blood and extracellular cystine levels during oxidative stress, such as the activation of glutathione and cysteine (promptly oxidized to cystine) efflux from the liver, skeletal muscles, and various tissues and organs. This occurs to counter the redox shift, as demonstrated in animal models [23]. A further source of cystine is the diet. In the subgroup of ASD children with food selectivity, we found −18.5% (*p* = n.s.) cysteine compared to children with a balanced diet. It can be argued that an excess of cystine may be due to impairments in the cystine/glutamate antiporter system x_c_^−^ [24,25,26]. Additionally, lactic acid discriminated between ASD and NT children (VIP = 0.88; *p* = 0.004), being higher in the former (+67%). This finding, in conjunction with the high levels of succinic acid (+69%, *p* = 0.048), suggests mitochondrial dysfunction [27], with the activation of the anaerobic glycolysis, the progressive impairment of astrocytes metabolism, the generation of intracerebral reactive oxygen species (ROS), and finally, irreversible astrocytes injury and cytolysis [28,29].

In the group of ASD children, the significant decrease in scylloinositol, a sugar derivative metabolite, may be considered an original finding: to our knowledge, this is the first time that this metabolic change was observed. High levels of scylloinositol in the brain of healthy subjects (approximately 100-fold greater than in the surrounding tissues) have been previously associated with a normal neurologic status [30,31]. Scylloinositol inhibits the aggregation of amyloid β peptide, improving several Alzheimer disease like-phenotypes. Notably, the depletion of scylloinositol has proconvulsant effects [32], and a reduction in scylloinositol may be consistent with an altered neurobiological profile.

Our results provide further insights into the specific changes of gut microbiota metabolites in autistic children [33]. First, the significant increase in urinary quinic acid, hippuric acid, tryptophan, and indole-3-acetic acid may be related to gut dysbiosis and dietary style [34,35]. Benzoic acid was also increased (+122%), although not significantly (*p* = n.s.). More importantly, we observed a significant increase in quinic acid (+182%; *p* = 0.027) in ASD children with food selectivity compared with those with a balanced diet. This difference may originate from the low abundance of *Lactobacillus* and *Bifidobacterium spp.* in children with food selectivity as the chlorogenic bacterial hydrolysis originates caffeic acid and quinic acid and the subsequent dehydroxylation and β-oxidation of the caffeic acid leads to benzoic acid [36,37]. Notably, in the subgroup with food selectivity we found a less pronounced increase in hippuric acid versus the subgroup with balanced diet (−62%; *p* = 0.017). The concomitant significant decrease in indole-3-acetic acid observed in the subgroup with food selectivity compared to balanced diet (−35%; *p* = 0.005) supports the assumption that the gut microbiota composition could be significantly different between the two subgroups. Second, at least two major pathways of the tryptophan metabolism were perturbed in ASD children. On the one hand, the direct metabolism of tryptophan by gut microorganisms led to a significant increase in indole-3-acetic acid, with effects on gut permeability and host immunity [38]. On the other hand, perturbations in the kynurenine pathway were confirmed by an increase in quinolinic acid (+36%, *p* = n.s.) and a decrease in kynurenic acid (−20%, *p* = n.s.) [39]. In the brain, quinolinic acid is a pro-inflammatory and pro-oxidant agent inducing excitotoxic effects [40], while kynurenic acid is an anti-oxidant, neuroprotective factor [41]. Finally, 5-hydroxyindoleacetic acid was significantly increased (+60%, *p* = 0.04) in ASD children, confirming the well-known activation of the serotonin pathway in enterochromaffin cells via tryptophan hydrolase 1 [42,43,44,45].

Diet influenced the role of 1-methylhistidine as a discriminant metabolite in ASD children: unlike 3-methylhistidine, which reflects both dietary intake and muscle catabolism, the majority of urinary 1-methylhistidine results from the enzymatic conversion of the anserine meat sources (especially poultry) into β-alanine and 1-methylhistidine [46]; in children with food selectivity, this increase was less pronounced (−24%, *p* = n.s.).

The importance of gut fermentation and dysbiosis in autistic children was finally confirmed by the discriminant role of allyl-thioacetic acid and leucine. Allylthioacetic acid may be derived from either the gut fermentation of vegetables by *Lactobacillus plantarum* [47] or by the metabolism of several yeasts and fungi [48]. Moreover, the increase in allylthioacetic acid may be related to a diet rich in cheese containing *Brevibacterium casei* [49]. Leucine, a branched-chain amino acid (BCAA), discriminated ASD from NT children; its higher urine level may be referred to the gut dysbiosis. In autistic subjects, the genera *Prevotella* and *Bacteroides* are less abundant than in non-autistic individuals [50]. Oddly, the species *Prevotella copri* and *Bacteroides vulgatus* are more represented in autism; both microorganisms are the main bacterial species contributing to blood and urine BCAA in humans [51,52,53]. Although not discriminant, valine was significantly increased in ASD children, independently of food selectivity (+32%, *p* = n.s.).

### 3.2. The Urine Metabolome Reflects Autism Core Symptoms Severity

Metabolomics provided robust evidence of the close relationship between the severity of ASD core symptoms, evaluated by ADOS-2 CSS, and the urine metabolic profile. ADOS-2 CSS is the gold standard for evaluating autistic symptoms, including social affect and repetitive, restricted behaviors [54]. In children with ADOS-2 CSS ≥8, the significant increase in 2-hydroxylacrylic acid, HPHPA, *p*-cresol, and tri hydroxypentanoic acid revealed the overgrowth of the genus *Clostridium* in the gut microbiota [55]. Specifically, 2-hydroxyacrylic acid, an unsaturated monocarboxylic acid derived from acrylic acid, is produced by *Clostridia spp.*, through the reduction of lactic acid to propionic acid via an acrylyl-CoA intermediate [56].

HPHPA is derived from phenylpropionic acid and mono hydroxylphenylpropionic acid; they are the products of the hydroxylation and deamination of dietary phenylalanine within the intestinal tract by multiple *Clostridia spp.*, including *C. perfrigens* and *C.* difficile [57]. Both precursors are converted to HPHPA by human metabolism; when the amount of HPHPA becomes chronically excessive, it can act as a neurotoxin and a metabotoxin. HPHPA excess is converted into hydroxyhippuric acid by the enzymes of the fatty acid oxidation, and our results confirmed an increase in 4-hydroxyhippuric acid (+128%; *p* = n.s.) in the subgroup of children with ADOS-2 CSS ≥ 8.

*C. difficile* and *Pseudomonas stutzeri* are the main sources of *p*-cresol in humans [58]; this organic aromatic compound acts as a neuroactive uremic toxin and is increased in autistic subjects [59]. Our results confirm that the magnitude of the *p*-cresol increase was proportional to the severity of ASD core symptoms, as previously demonstrated in both humans and animal models [60,61], and is associated with slow bowel transit due to the decrease of gut motility [62].

Trihydroxypentanoic acid is derived from pentanoic (valeric) acid, a short-chain fatty acid commonly found in human feces and produced by various *Clostridia spp.* and other gut bacteria via the condensation of ethanol with propionic acid [63]. In our ASD children, a significant positive correlation was found between ADOS-2 CSS and 2-hydroxylacrylic acid, HPHPA, and trihydroxypentanoic acid (Table S3).

Two dicarboxylic acids, adipic acid and oxalic acid, significantly discriminated between children with mild-to-moderate ASD core symptoms and those with severe ASD core symptoms, and were found increased in the latter subgroup by 3.9- and 1.4-folds, respectively. High urinary adipic acid levels in autistic subjects may be associated with excess intake of food containing adipic acid (e.g., puddings, fruit gels, no-bake cream pies) and alterations in mitochondrial β-oxidation of fatty acids and, in turn, impaired ω-oxidation [64,65]. In animal models of autism, it was demonstrated that adipic acid inhibits the activity of two enzymes: L-glutamate decarboxylase and GABA transaminase [66,67]. The validity of our findings is supported by similar results previously published in the literature [68] and by the significant positive correlation between ADOS-2 CSS and adipic acid level (ρ = 0.50; *p* = 0.02).

Children with severe ASD core symptoms showed a significant increase in palmitic acid; in the whole group of autistic children, palmitic acid significantly correlated with ADOS-2 CSS (ρ = 0.65; *p* = 0.03). This long-chain saturated fatty acid, also known as hexadecanoic acid, comes from the diet or de novo lipogenesis in the liver and can easily cross the blood–brain barrier. Within the CNS, palmitic acid activates the transmembrane toll-like receptor 4 (TLR4), which in turn activates microglial cells, triggering a strong inflammatory cascade [69]. Moreover, palmitic acid induces ROS production leading to the inhibition of diglyceride acyltransferase (DGAT2) activity [70,71,72].

The significant increase in 1-deoxypentitol in the subgroup of children with severe ASD core symptoms may originate from both the diet and the growth of fungi and yeasts in the intestinal tract [73]. Briefly, 1-deoxypentitol is a derivative of pentitol, also known as arabitol or arabinitol. In autistic patients, previous studies found a significant increase in urinary pentitol [74]; this finding was associated with the overgrowth of *Candida spp.* [75]. Our results confirm previous findings and are supported by the close correlation between 1-deoxypentitol and ADOS-2 CSS (Table S3). Based on the robustness of our data, the confirmation of data previously published in the literature, and the correlation between clinical scores and metabolite levels, we consider at least three metabolites to be candidate urine biomarkers of ASD core symptoms severity: HPHPA, adipic acid, and palmitic acid.

### 3.3. Metabolomics Contributes to Identifying Clinical Thresholds Discriminating Severe from Moderate Behavioral Impairments

The agglomerative HCA, followed by the OPLS-DA model, clearly demonstrated that, in our autistic cohort, inter-individual metabolomic heterogeneity could be grouped into two highly distinguished metabolic clusters. Cluster II represents a homogeneous metabolic profile belonging to approximately 85–90% of children with mild-to-moderate behavioral impairment, evaluated by the RBS-R and ABC-C questionnaires. In cluster I, children with severe behavioral impairment were less represented (approximately 54–64%), suggesting that a score threshold discriminating children with severe restricted, repetitive behaviors is currently unlikely. The small size of cluster I (n = 11) may be considered a co-factor in this inconclusive result. However, the metabolic and clinical phenotypes were identified by different approaches: the former results from the objective recognition of metabolites by standardized analytical and statistical methods, namely metabolomics, while the latter results from the subjective observation of children’s behaviors by parents, despite the standardization of questionnaires. Therefore, we postulate that metabolomics may be more accurate than questionnaires filled by parents.

### 3.4. Limitations of the Study

Our study has several limitations. First, the number of enrolled ASD and NT children was quite small; thus, we cannot create a definitive model for ASD prediction. Second, in ASD children, gastrointestinal disease and dietary habits were not evaluated by standardized specific tools; in addition, NT children were not evaluated by ADOS-2 CSS, even though their inclusion as a control group was based upon parental report and clinical observation by trained clinicians with deep expertise on autism. Third, neither a food diary recording specific daily dietary habits was utilized in our study, nor height, weight, and body mass index data were stored. Fourth, we performed neither metagenomic nor a culture-based analysis of the gut microbial flora. However, the identity of the gut microbiota is reflected by bacterial metabolites [76,77] and the urine metabolome is strongly influenced by interactions between gut microbial metabolites migrating into the circulation with their targets [78]. Strengths of this study include the rigorous control of the standardization of all the variables associated with clinical evaluation, therapeutic treatment, and preanalytical steps (samples collection, transport, and storage); the evidence that changes in the urine metabolic profile of ASD children reflect differences in ASD severity; the finding that in our ASD children, the urine metabolic profile reflect two metabolic subgroups, each of them associated with a clinical score range computed by the RBS-R and ABC-C scales.

## 4. Materials and Methods

### 4.1. Participants

Fifty-seven Italian children aged 2–11 years were enrolled in the study: 31 (23 males) subjects with idiopathic ASD and 26 NT children (16 males) matched for age and ethnicity with ASD children. All the autistic children came from families with no considerable social class differences between each other. The study protocol was approved, registered (R.S. #146/16), and monitored by the local institutional review board. Informed consent from a parent or legal guardian was obtained for each participant. Diagnosis of ASD was established following the Diagnostic and Statistical Manual of Mental Disorders 5^th^ Edition DSM-5 criteria [79]. All the autistic children underwent a clinical assessment, developmental history and a comprehensive evaluation of developmental level (Psychoeducational Profile Third Edition—PEP-3). The evaluation of gastrointestinal disease in ASD children consisted of recognizing chronic constipation, abdominal pain, chronic diarrhea, and gastroesophageal reflux. No autistic child enrolled in our study had clinical and laboratory signs of kidney dysfunction. Exclusion criteria were known inborn errors of metabolism or suspected genetic syndromes, neurological syndromes or focal neurological signs, anamnesis of severe birth asphyxia, head injury or epilepsy, and ongoing acute diseases. NT children were selected from a pediatric primary healthcare service placed within the district area of the University-Hospital Tor Vergata; they were primarily examined to rule out any genetic background of a family history of autism. Then, they underwent clinical and observational assessments performed by trained clinicians with expertise in autism. Parents of NT children filled in the Child Behavior Checklist (CBCL) questionnaire, to exclude the presence of atypical and problematic behaviors. Further exclusion criteria for NT children included: a history of maternal substance abuse during pregnancy; neurological and psychiatric disorders; ongoing acute diseases; major physical abnormalities, and the sensory deficiency (e.g., blindness, deafness); known inborn errors of metabolism.

### 4.2. Primary Behavioral Outcome Measures in Autistic Children

ASD core symptoms were evaluated by the ADOS-2 performed by a licensed clinician [80,81]. The ADOS-2 consists of 5 independent modules, administered on the basis of expressive language level and age; it provides a specific measure for the level of autism severity, the Calibrated Severity Score (CSS). The CSS ranges from 1 to 10, identifying 4 different categories of symptoms severity: 1–2 none; 3–4 mild; 5–7 moderate; 8–10 high. Repetitive and restricted interests were estimated by the Repetitive Behavior Scale-Revised (RBS-R) [82], and problematic behaviors by the ABC-C parental questionnaire [83,84]. RBS-R is a 43-item questionnaire organized in six subscales: stereotypic behavior; self-injurious behavior; compulsive behavior; ritualistic behavior; sameness behavior; restricted interests. A total score was obtained by combining results from subscales; the higher the score the greater the severity of behavioral impairments. We used a five-factor solution scoring, which implies that the ritualistic behavior and sameness behavior subscales are integrated each other [85]. ABC-C is a parental questionnaire consisting of 58 items grouped in five subscales: irritability; social withdrawal; stereotypic behavior; hyperactivity/noncompliance; inappropriate speech. A total score was computed for each ASD child; the higher the score the greater the severity of behavioral impairments.

### 4.3. Sample Collection, Storage, and Preparation

First-morning urine samples were collected by parents at home within sterile bags and delivered to the Children Psychiatric Unit of the University Hospital of Tor Vergata before the end of that morning. Samples were immediately centrifuged; supernatants were transferred into cryo-vials, frozen and stored at −25 °C until their shipping to the metabolomics laboratory. Samples transportation was organized by using storage boxes placed inside a biohazard bag containing dry ice. The length of samples transportation from Tor Vergata (Rome) to Monserrato (Cagliari) did not exceed five hours; during this interval, samples temperature constantly remained below −20 °C, as recorded by a data logger. Ultimately, samples were stored within the metabolab at −45 °C until analysis. Samples were pre-treated and analyzed simultaneously; a partially modified standardized protocol for sample preparation was applied [86].

### 4.4. Gas Chromatography-Mass Spectrometry (GC-MS) Analysis

Derivatized samples were analyzed by a global unbiased mass spectrometry-based platform incorporating an Agilent 5975C interfaced to the GC 7820 (Agilent Technologies, Palo Alto, CA, USA). The system was equipped with a DB-5 ms column (Agilent J&W Scientific, Folsom, CA, USA); the injection temperature was set at 230 °C, and the detector temperature at 280 °C. The carrier gas Helium flow rate was 1 mL/min. The deconvolution of all raw spectra into a data matrix was made by the Automated Mass Spectral Deconvolution and Identification System (AMDIS) software, available at http://chemdata.nist.gov/mass-spc/amdis (last access, 28 July 2020). Metabolites were identified by comparing retention times, and mass spectra with those stored in an in-house made library including more than 255 metabolites. Further metabolites were identified by using the National Institute of Standards and Technology mass spectral database (NIST08) [87] and the Golm Metabolome Database [88], available at http://gmd.mpimp-golm.mpg.de (last access, 14 August 2020). The relative intensity of each peak was normalized against the internal standard (98% Heptadecanoic acid) in GC–MS run. We pooled an aliquot from all samples to obtain a composition of the quality control (QC) sample, reflecting the aggregate metabolite composition of all of our biological samples [89,90]. Multiple aliquots of QC were then prepared and stored. Samples were analyzed in a single batch; at first, QC aliquots were injected five times to ensure system stability. The sequence of QC aliquots was arranged according to published guidelines [91].

### 4.5. Data Processing and Statistical Analysis

Results were normalized by urinary specific gravity [92], taking into account that creatininuria may be significantly reduced in random urine samples of ASD children [93]. Data were at first normalized by sum, logarithmic transformation, and auto-scaling, according to Pareto [94,95]. Univariate statistical analysis included the non-parametric Mann-Whitney U test and the Spearman’s rho (ρ) correlation test. The non-parametric Mann-Whitney U test evaluated differences in urine metabolite levels between groups; *p* < 0.05 was considered statistically significant. The Spearman’s rho (ρ) correlation test evaluated the correlation between metabolite level and clinical score; the closer to zero, the weaker is the association between ranks. Orthogonal projection to latent structure-discriminant analysis (OPLS-DA), supported by the SIMCA-P+ software (version 14.1 Umetrics AB, Umeå, Sweden), was used to reducing model complexity, making sample discrimination more straightforward. The validity of the OPLS-DA model was assessed by the cumulative modeled variations in the X and Y matrixes (R^2^ X and R^2^ Y, respectively) and by the cross-validated predictive ability Q^2^. To evaluate the significance of the model, the cross-validation analysis of variance (CV-ANOVA) was applied [96]. The optimal model performance was tested by the receiver operating characteristic (ROC) analysis, yielding the area under the curve (AUC) value, and by the validation data set, using MetaboAnalyst 4.0 [97]. By supervised analysis, we obtained the set of variables importance in projections (VIP); variables were selected by computing the VIP score for each variable and ruling out all the variables with the VIP score below 1, a predefined threshold [98]. Network analysis was applied to describe the properties of metabolites and their complex structural and biochemical relationships [99]. Metabolic networks were mapped by the Data Analysis and Visualization engine (DAVe) software, available at https://creative-data.science/dave/, last access 31 August 2020, in conjunction with the Kyoto Encyclopedia of Genes and Genomes (KEGG) and PubChem CID identifiers, National Center for Biotechnology Information (NCBI), PubChem compound database available from: http://pubchem.ncbi.nlm.nih.gov) [100,101]. Metabolite relationships resulted in a certain number of nodes (variables) and edges (relationships). Network analysis elucidates the complex biochemical alterations correlated with changes in physiological and pathological conditions. The MetaMapR statistical programming language and environment, the ancillary software implemented in R (v.3.0.1), was also used [102]. MetaMapR combines biochemical relationships with structural similarity, mass spectral similarity, and correlations, treating all enzymatic relationships indirectly. The structural similarity was determined using both similarities between PubChem Substructure Fingerprints, available from ftp://ftp.ncbi.nlm.nih.gov/pubchem/specifications/pubchem_fingerprints.txt, last access 29 July 2020, and Tanimoto coefficients, calculated for each metabolite. The network structural similarity threshold was set to a Tanimoto score ≥ 0.7; variables with a score ≤ 0.7 were removed. DAVe and MetaMapR software functionalities were used to load ASD datasets: subjects were listed in rows, and metabolites in columns. The DAVe network module provides statistical tools to calculate a variety of networks, including correlation, biochemical relationships, and structural similarity networks. The resulting networks were visualized by Cytoscape [103]. The Ward’s hierarchical clustering analysis (HCA) was used to classify the urine metabolomes of ASD children into homogeneous classes [104,105]. HCA incorporates a succession of sequential steps merging variables into homogeneous clusters; at each step, a new cluster is formed with the aim to increase both within-group homogeneity and between-groups heterogeneity. In this study, we used the average linkage, also referred to as the Unweighted Pair-Group Method using Arithmetic averages (UPGMA), because it is a natural compromise between single linkage and complete linkage, as it is sensitive to the shape and size of clusters. The HCA algorithm was visualized using a dendrogram, a tree-like diagram that records the sequences of merges or splits.

## 5. Conclusions

The early diagnosis and therapeutic intervention improve long-term outcomes in ASD. Although the American Academy of Pediatrics recommended ASD screening at 18- and 24-month visits, the average age of autism diagnosis is still around four years of age [106]. Our findings open up new perspectives for a better understanding of the correlation between the clinical phenotype of autistic children and their urine metabolome. The severity of ASD core symptoms and problematic behaviors may be associated with specific metabolic perturbations, most of them induced by an overgrowth of *Clostridia spp.*, changes in the gut mycobiome (e.g., overgrowth of *Candida sp.*), and by alterations in mitochondrial functions. Further studies on larger cohorts are required to confirm our results and identify a urinary metabolomic fingerprint characterized by higher specificity and sensitivity for ASD.

## Figures and Tables

**Table 1:** Basic demographic and clinical characteristics of 31 children with autism spectrum disorder (ASD). Median and IQR were computed from results obtained in 31 ASD children.

Parameter	Median (IQR) ^1^	Range
Age (y)	5 (3–6)	2–11
Gender (M/F)	23/8	===
Family type (n simplex/n multiplex)	25/6	===
Gastrointestinal disease (n GI/n total)	6/31	===
Food selectivity (n FS/n total)	17/31	===
Developmental Level	50 (43–60)	21–84
ADOS-2 CSS ^2^	7.5 (6–9.2)	3–10
SCQ score ^3^	15 (11–22)	2–42
SRS score ^4^	78 (70–90)	50–101
RBS-R score ^5^	23 (8.5–35)	2–94
ABC-C score ^6^	40 (23–55)	4–128

^1^ IQR, interquartile range. ^2^ ADOS-2 CSS, Autism Diagnostic Observation Schedule second Edition with the Calibrated Severity Score; ^3^ SCQ, Social Communication Questionnaire; ^4^ SRS, Social Responsive Scale; ^5^ RBS-R, Repetitive Behavior Scale-Revised; ^6^ ABC-C, Aberrant Behavior Checklist-Community.

**Table 2:** Scores resulting from clinical assessments and behavioral scales tested for the ASD child #69. Median and IQR were computed from results obtained in 31 ASD children.

Clinical Assessment	Score		
	Result	Median (IQR) ^1^	Range
Age (y)	5.8	5 (3–6)	2–11
Gastrointestinal disease	No	===	===
Food selectivity	Yes	===	===
Developmental level	43	50 (43–60)	21–84
ADOS-2 CSS ^2^	6	7.5 (6–9.2)	3–10
SCQ ^3^	11	15 (11–22)	2–42
RSR ^4^	71	78 (70–90)	50–101
RBS-R ^5^	15	23 (8.5–35)	2–94
ABC-C ^6^	18	40 (23–55)	4–128

^1^ IQR, interquartile range. ^2^ ADOS-2 CSS, Autism Diagnostic Observation Schedule second Edition with the Calibrated Severity Score; ^3^ SCQ, Social Communication Questionnaire; ^4^ SRS, Social Responsive Scale; ^5^ RBS-R, Repetitive Behavior Scale-Revised; ^6^ ABC-C, Aberrant Behavior Checklist-Community.

**Table 3:** List of the most significant metabolites, obtained by univariate and multivariate statistical analysis, discriminating children with autism spectrum disorder (ASD) from neurotypical (NT) children.

Metabolite	Two-Tailed Mann Whitney U test		VIP *	% Difference ASD vs. NT
	*p*	*z*-Score		
7-Methylxanthine	0.012	2.48	3.30	−61%
Scylloinositol	0.011	2.52	2.43	−35%
Uric acid	0.002	−3.02	2.41	−50%
Aminomalonic acid	0.034	2.10	1.73	−52%
Quinic acid	0.002	3.11	1.72	+263%
Hippuric acid	0.003	−2.93	1.65	+164%
Tryptophan	0.024	2.25	1.44	+100%
1-Methylhistidine	0.015	−2.42	1.41	+67%
Cystine	0.018	2.36	1.37	+101%
Indole-3-acetic acid	0.036	2.10	1.20	+61%
Allyl thioacetic acid	0.014	−2.46	1.12	+28%
Leucine	0.006	2.76	0.93	+49%
Lactic acid	0.004	−2.87	0.88	+67%

* VIP, variable importance of projection.

**Table 4:** List of the most significant metabolites, obtained by univariate and multivariate statistical analysis, discriminating ASD children with severe autism spectrum disorder core symptoms (ADOS-2 CSS ≥ 8) from those with mild-to-moderate core symptoms (ADOS-2 CSS < 8).

Metabolite	Two-Tailed Mann Whitney U Test		VIP *	% Difference ASD vs. NT
	*p*	*z*-Score		
2-Hydroxyacrylic acid	0.002	3.09	2.27	+321%
Sucrose	0.009	2.58	1.89	+234%
Allantoin	0.003	3.00	1.85	+143%
3-Methylhistidine	0.039	2.06	1.84	+726%
Adipic acid	0.005	2.83	1.80	+389%
3-(3-Hydroxyphenyl)-3-hydroxypropanoic acid	0.008	2.64	1.74	+142%
Xylose	0.002	3.06	1.69	+364%
1-Deoxypentitol	0.0002	−3.68	1.68	+294%
Glyceric acid	0.016	2.41	1.65	+265%
Palmitic acid	0.005	2.80	1.63	+263%
Hippuric acid	0.044	2.00	1.60	+196%
Homovanillic acid	0.011	2.54	1.58	+204%
5-Hydroxyindoleacetic acid	0.011	2.54	1.48	+131%
Ribitol	0.003	3.00	1.47	+156%
Benzoic acid	0.84 *	1.73 *	1.42	+266%
Proline	0.003	3.00	1.41	+148%
*p*-Cresol	0.048	−1.97	1.40	+105%
Quinolinic acid	0.004	2.84	1.39	+262%
Lactic acid	0.009	2.59	1.26	+88%
Oxalic acid	0.035	2.11	1.22	+138%
Mannose	0.010	2.56	1.12	+204%
Trihydroxypentanoic acid	0.007	2.68	1.08	+96%

* VIP, variable importance of projection.

**Table 5:** Statistical parameters of the OPLS-DA models derived from the GC-MS spectra of urine samples. Autistic children with severe abnormal behaviors (either with RBS-R or ABC scores above the cut-off limit) were compared with autistic children with mild-to-moderate abnormal behaviors (either with RBS-R or ABC scores below the cut-off limit).

Screening	Candidate Cut-Off Level	R^2^ X	R^2^ Y	Q^2^	P
RBS-R	>35	0.496	0.933	0.557	1.00
ABC-C	>50	0.487	0.822	0.465	0.026

Cut-off level: candidate threshold discriminating severe from mild-to-moderate abnormal behaviors. RBS-R: Repetitive Behavior Scale-Revised. ABC-C: Aberrant Behavior Checklist-Community. R^2^ X: direction of the maximum covariance between a dataset. R^2^ Y: class membership. Q^2^: cross-validated predictive ability. P: cross-validation analysis of variance.

## Supplementary Materials

The following are available online at https://www.mdpi.com/2218-1989/10/11/476/s1, Supplementary Materials includes: Table S1: List of metabolites ruled out from statistical analysis, Table S2: List of unknown metabolites recognized in this study by GC-MS, Table S3: List of urine metabolites significantly correlating with scores computed by the Diagnostic Observation Schedule Second Edition with Calibrated Severity Score (ADOS-2 CSS), Table S4: Data matrix combining scores from RBS-R and ABC questionnaires with urine metabolome of ASD children, Figure S1: ROC plot referred to the comparison between typically developed children and ASD children, Figure S2: OPLS-DA score plot comparing the urine metabolic profile of the subgroup of ASD children with ADOS-2 CSS score ≥8 (blue dots) and that of ASD children with ADOS-2 CSS score < 8 (red dots). The robustness of the model is supported by R^2^ X = 0.513, R^2^ Y = 0.722, Q^2^ = 0.468 and confirmed by the analysis of variance CV-ANOVA (*p* = 0.0092).
