# Supplementary material for: The Urine Metabolome of Young Autistic Children Correlates with Their Clinical Profile Severity
Source: Metabolites. 2020 Nov 23;10(11):476. doi: 10.3390/metabo10110476 (PMC7700197; doi:10.3390/metabo10110476)
Supplement: Supplementary file 1 [file metabolites-10-00476-s001.pdf]

The urine metabolome of young autistic children correlates with the severity of clinical profile.
Supplementary Materials

Table S1. List of metabolites ruled out from statistical analysis

Valero lactam
5-Hydroxyhexanoic acid
3-Methylglutaconic acid
Ornithine lactam
Malic acid
Taurine
Anhydroglucose
3-Phosphoglycerate
Fructose
Chiroinositol
N-Acetylgalactosamine

Table S2. List of unknown metabolites recognized in this study by GC-MS.

Unknown 358 U	Sugar 1382
Unknown 794 U	Unknown 1457 U
U 807	Sugar 1602
Acid 823	Sugar 1624
Unknown 835 U	Monosacch E
Unknown 906 U	Sugar 1646
U D	U C
U 972	A231002
U 987	Monosacch 1886
A160001	U A
Unknown 1089 U	Unknown 1910
Sugar 1369	Disaccharide
A201005	

Table S3. List of urine metabolites significantly correlating with scores computed by the Diagnostic Observation Schedule Second Edition with Calibrated Severity Score (ADOS-2 CSS)

METABOLITE	SPEARMAN'S CORRELATION	
	ρ	*p*
2-Hydroxyacrylic acid	0.46	0.028
Allantoin	0.44	0.025
Adipic acid	050	0.023
3-(3-Hydroxyphenyl)-3-hydroxypropanoic acid	0.46	0.020
Xylose	0.45	0.021
1-Deoxypentitol	0.45	0.020
Palmitic acid	0.65	0.003
Lyxonic acid	0.38	0.044
Quinolinic acid	0.43	0.033
Tri hydroxypentanoic acid	0.40	0.033

Table S4. Data matrix combining scores from RBS-R and ABC questionnaires with urine metabolome of ASD children

URINE METABOLOME IDENTIFICATION	SCORE		URINE METABOLOME IDENTIFICATION	SCORE	
	RBS-R	ABC		RBS-R	ABC
Cluster 1 (n=11)			***Cluster 2 (n=20)***		
#69	15	18	#46	34	50
#27	24	80	#34	47	38
#61	65	70	#4	24	44
#28	51	59	#21	33	48
#37	9	34	#6	24	42
# 2	3	44	#23	35	50
#75	43	54	#30	8	17
#19	94	128	#55	17	32
#22	38	75	#33	31	81
#49	36	51	#67	19	45
#8	2	12	#58	35	23
			#15	36	46
			#11	12	23
			#85	4	10
			#60	39	94
			#13	2	4
			#71	35	49
			#39	6	49
			#7	23	38
			#10	23	29

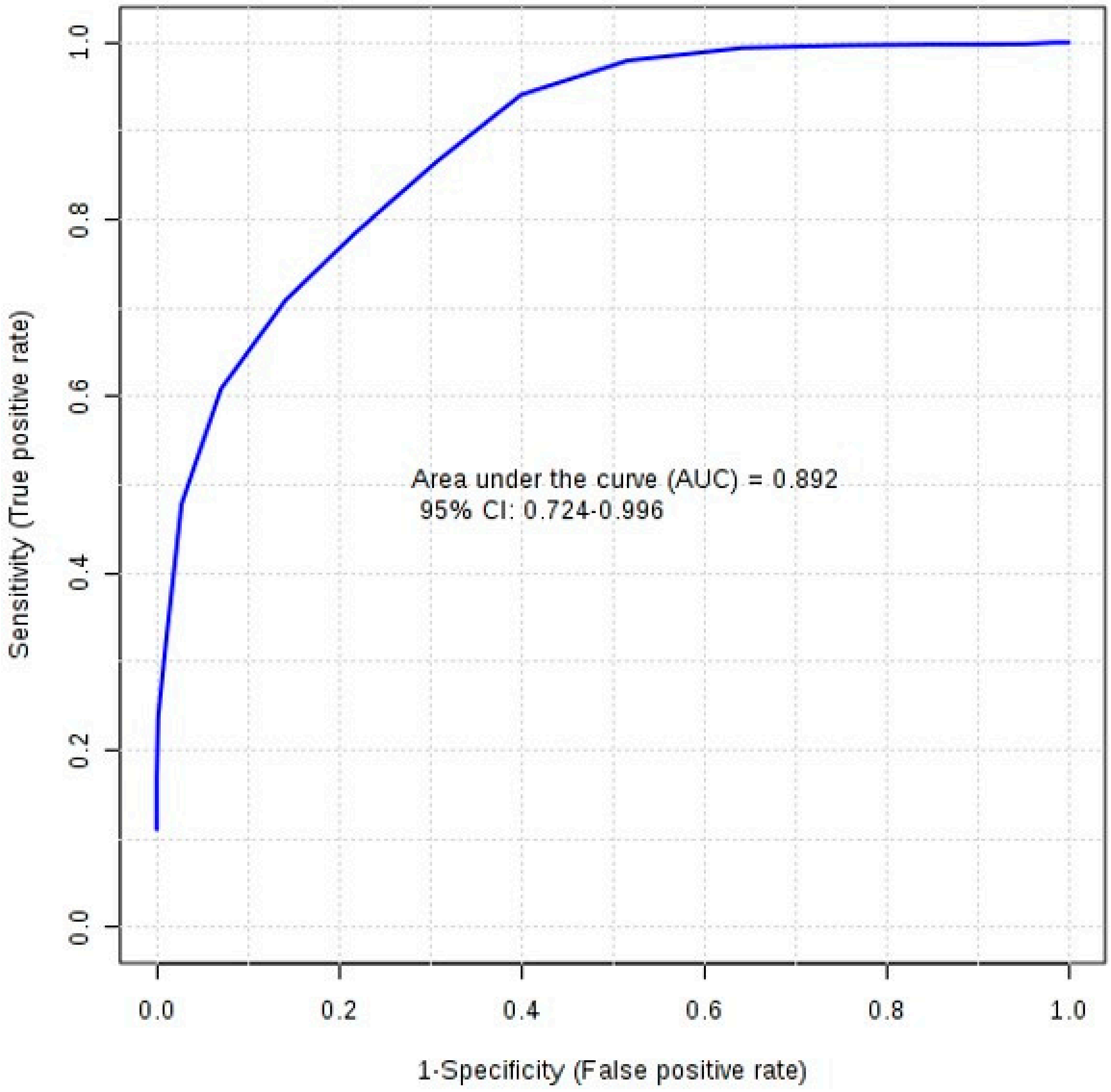

Figure S1. ROC plot referred to the comparison between typically developed children and ASD children

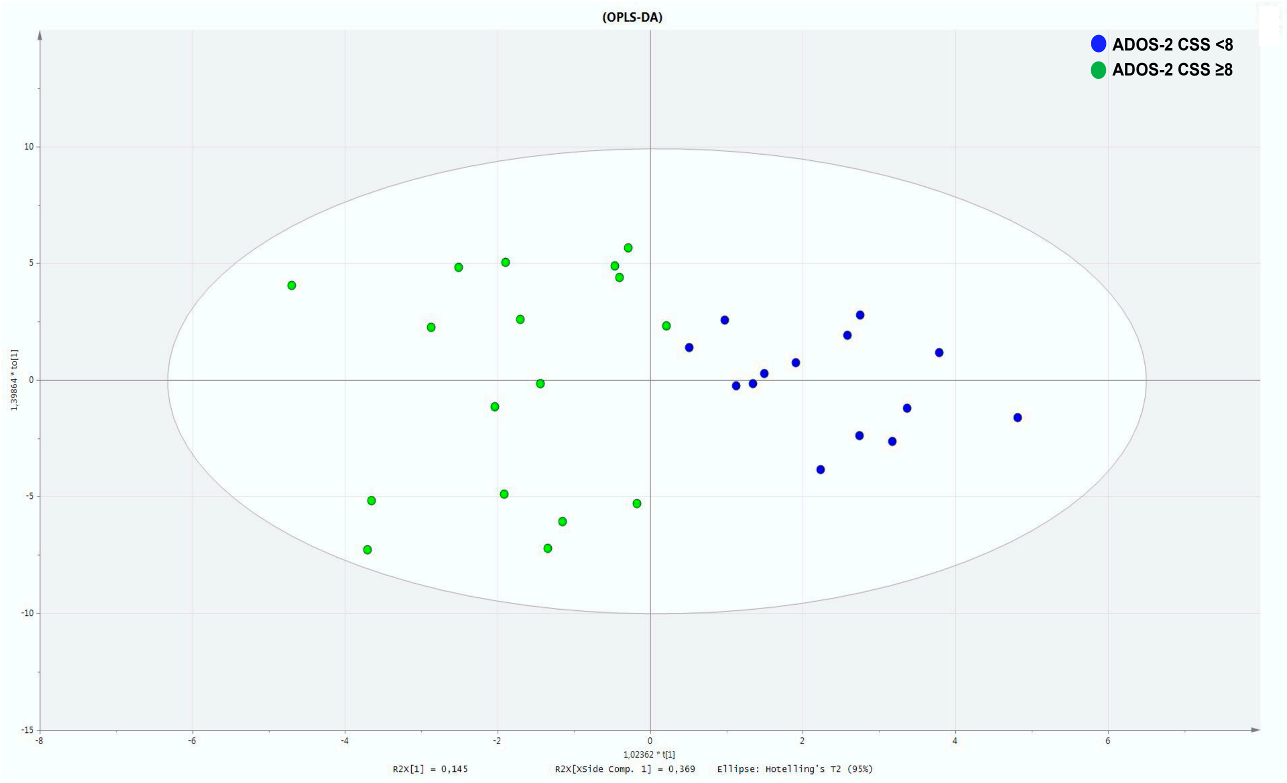

Figure S2. OPLS-DA score plot comparing the urine metabolic profile of the subgroup of ASD children with ADOS-2 CSS score ≥8 (blue dots) and that of ASD children with ADOS-2 CSS score <8 (red dots). The robustness of the model is supported by R^2 X = 0.513, R^2 Y = 0.722, Q^2 = 0.468 and confirmed by the analysis of variance CV-ANOVA (p=0.0092).
